# Identifying key bioprocess variables using explainable machine learning to enhance culture efficiency and viability of umbilical cord-derived mesenchymal stem cells

**DOI:** 10.7150/ijms.127764

**Published:** 2026-03-30

**Authors:** Tse-Pu Huang, Hsin-Hui Huang, Bing-Tsiong Li, Pei-Hung Shen, Gracy Thomas, Juin-Yi Han, Chi-Ming Chu, Kun-Yi Lin

**Affiliations:** 1 Department of Orthopedic Surgery, Tri-Service General Hospital, National Defense Medical University, Taipei, Taiwan.; 2 Department of Biotechnology and Laboratory Science in Medicine, National Yang Ming Chiao Tung University, Taipei, Taiwan.; 3 Graduate Institute of Linguistics, National Cheng Chi University, Taipei, Taiwan.; 4 Institute of Communications Engineering, National Tsing Hua University, Hsinchu, Taiwan.; 5 Graduate Institute of Technology, Innovation and Intellectual Property Management, National Cheng Chi University, Taipei, Taiwan.; 6 School of Public Health, National Defense Medical University, Taipei, Taiwan.; 7 Department of Public Health, China Medical University, Taichung, Taiwan.; 8 Graduate Institute of Life Sciences, National Defense Medical University, Taipei, Taiwan; 9 Graduate Institute of Medical Sciences, National Defense Medical University, Taipei, Taiwan.; 10 Big Data Research Center, College of Medicine, Fu-Jen Catholic University, New Taipei City, Taiwan; 11 Department of Public Health, College of Health Sciences, Kaohsiung Medical University, Kaohsiung, Taiwan; 12 Department of Healthcare Administration and Medical Informatics, College of Health Sciences, Kaohsiung Medical University, Kaohsiung, Taiwan; 13 Department of Medical Research, Kaohsiung Medical University Hospital, Kaohsiung, Taiwan

**Keywords:** umbilical cord-derived mesenchymal stem cell, XGBoost algorithm, culture duration, cell viability

## Abstract

**Background:**

Human umbilical cord-derived mesenchymal stromal/stem cells (UC-MSCs) are promising for regenerative medicine, but consistent manufacturing quality is critical.

**Objective:**

To develop and interpret machine-learning models (Extreme gradient boosting (XGBoost), with Shapley Additive Explanations, SHAP) that identify facilitatory and inhibitory factors affecting UC-MSC culture duration and post-processing viability.

**Methods:**

We analyzed data from 203 UC-MSC manufacturing cases. Candidate predictors included neonatal characteristics (e.g., sex, delivery mode), processing timelines, medium composition, cell features, and operator-related factors. Performance was evaluated using accuracy, the area under the receiver operating characteristic curve (AUROC), the area under the precision-recall curve (AUPRC), log loss, and Brier score, with calibration assessed in cross-validation.

**Results:**

For predicting shorter culture duration (defined as a time interval between UC collection and the completion of cryopreservation of <600 h), the model achieved accuracy = 0.80, AUROC = 0.72, and log loss = 0.55; cross-validation yielded AUROC = 0.68, AUPRC = 0.81, and Brier score = 0.20 with good calibration. For predicting higher cell viability, the model achieved accuracy = 0.71, AUROC = 0.72, and log loss = 0.62; cross-validation yielded AUROC = 0.54, AUPRC = 0.58, and Brier score = 0.26. SHAP analysis indicated that shorter culture duration was most associated with medium composition, processing time, and delivery mode, whereas higher viability was linked to neonatal sex, operator identity, and processing time. Sensitivity analyses showed stable top-ranked features across decision-threshold shifts and after removing operator identity.

**Conclusions:**

An interpretable XGBoost+SHAP pipeline is effective for identifying process-critical drivers of UC-MSC culture duration. While current predictive precision for cell viability remains limited, the framework functions as a robust diagnostic tool for elucidating qualitative trends. By exploiting these insights, the model facilitates targeted optimization of media selection, timeline control, and standard operating procedures (SOPs), ultimately enhancing manufacturing quality.

## Introduction

Mesenchymal stromal/stem cells (MSCs) are promising candidates for cell therapy because of their regenerative, immunomodulatory, and trophic properties 1. MSCs can be isolated from multiple tissues, including bone marrow, adipose tissue, and the human umbilical cord (UC) 2-4. Among these, umbilical cord-derived mesenchymal stromal/stem cells (UC-MSCs), largely originating from Wharton's jelly, offer practical advantages, namely, noninvasive procurement, abundant supply, high proliferative capacity, broad differentiation potential, and low immunogenicity, increasing their preclinical and clinical use 3-6. Because therapeutic benefit is closely tied to product quality, UC-MSC quality should be consistent across the manufacturing continuum, from postpartum UC isolation and primary culture to expansion and precryopreservation, for clinical translation.

UC-MSC quality is influenced by various preanalytical and in-process variables: neonatal and obstetric factors, logistics/handling timelines, medium composition, and operator-dependent execution. The composition of culture media, which provide essential nutrients and bioactive cues, can affect growth kinetics and phenotypes 7,8. Obstetric conditions, such as gestational diabetes, maternal obesity, prematurity, and preeclampsia, have been shown to alter UC-MSC characteristics, including their differentiation potential, proliferation, and viability 9-13. Despite these insights, the joint, potentially nonlinear effects between neonatal factors (e.g., sex, birth weight, and delivery mode) and time-resolved processing parameters remain underexplored at the manufacturing scale.

Design of experiments is a powerful tool in bioprocess development but is constrained by factorial growth, use of prespecified models, and limited ability to capture complex interactions in cases with many covarying variables 14. By contrast, modern machine learning (ML) approaches can model high-dimensional, structured data; discover nonlinear patterns; and rank features without strong a priori assumptions; these advantages have driven the adoption of ML techniques across biotechnology and systems biology 15-18. Extreme gradient boosting (XGBoost) is a data-efficient tree-based ensemble that handles missingness and captures higher-order interactions—features suitable for heterogeneous manufacturing datasets 17,18.

However, black-box predictions have limited utility in regulated biomedical contexts, where predictions should be interpretable to practitioners. Explainable artificial intelligence (XAI) addresses this gap. Several XAI techniques can be applied to XGBoost, including partial dependence plot (PDP), accumulated local effect (ALE), and local surrogate methods such as local interpretable model-agnostic explanations (LIME). While these methods provide useful insights, they exhibit important limitations, including assumptions of feature independence (e.g. PDP), sensitivity to data distribution or sampling variability (e.g. LIME), and challenges in delivering explanations that are both locally faithful and globally consistent, particularly in the presence of complex feature interactions 19. Shapley additive explanations (SHAP) provide theoretically grounded, locally accurate decompositions of predictions into additive feature contributions, enabling global importance ranking and case-level rationale inspection 20. In perioperative and broader medical applications, SHAP improves transparency and supports clinician trust and decision-making by revealing variables driving model outputs 15,16,20.

Here, we present an explainable ML framework that integrates XGBoost with SHAP to interrogate real-world UC-MSC manufacturing data. We considered two critical quality indicators: (i) culture duration (shorter duration as a proxy for efficient expansion with mitigated risk of prolonged *in vitro* senescence) and (ii) cell viability (quantification of 7-aminoactinomycin D [7-AAD] via flow cytometry) 21,22. Using 203 consecutive cases, from UC isolation to cryopreservation, we trained and evaluated models to identify facilitatory and inhibitory factors: neonatal characteristics, logistics timelines, medium components/brands, immunophenotypes, and operator identity. We aim to deliver actionable, interpretable insights to optimize UC-MSC bioprocesses and support data-driven quality systems.

## Materials and Methods

### Data collection

This paper is a retrospective study that was approved by the Institutional Review Board. A total of 203 participants were enrolled in this study. Following the birth of each newborn, the UC was separated and UC-MSCs were cultivated. Samples were excluded if they failed to meet UC-MSC storage eligibility, such as those containing microorganisms, including yeast, mycoplasma, or bacteria, or if UC blood tested positive for infectious diseases, including hepatitis B virus (HBV), hepatitis C virus (HCV), syphilis, human T-lymphotropic virus (HTLV), or cytomegalovirus (CMV). UC-MSC quality was optimized before long-term cryopreservation. A comprehensive dataset encompassing UC isolation to UC-MSC cryopreservation was compiled, including birth weight, birth method, infant sex and blood type, length of navel cord, operation time, operator, and cell medium composition. The quality of UC-MSCs was assessed based on two indices: culture duration and cell viability. Culture duration was the time interval between UC collection and the completion of cryopreservation. Cell viability was evaluated by quantifying 7-AAD expression 22. This value was expressed as 100% minus the percentage of 7-AAD-positive cells. Cell quality was considered acceptable if the culture duration was <600 h or the cell viability was 99% or higher. Due to the retrospective nature of this study, no patients were involved in experimental design, implementation, or reporting, so the requirement for informed consent was waived.

### Model development and data preprocessing

Data preprocessing, model establishment, and parameter identification were sequentially conducted to investigate the facilitatory and inhibitory factors affecting UC-MSC quality. The data were preprocessed to ensure the dataset was structured, consistent, and optimized for training an effective ML model. Data not contributing to the evaluation of cell quality outcomes or not representing post cultivation information were deemed irrelevant and removed. Missing values in numerical variables were imputed using the median of the training dataset, while categorical features were imputed using the most frequent value. The dataset was standardized by converting timestamps related to UC processing and UC-MSC cultivation into floating-point representations of elapsed time. Numerical variables, including the transformed elapsed-time data, were normalized to maintain consistency across different scales. Specifically, all numeric variables were standardized using z-score normalization, where each value was rescaled by subtracting the mean and dividing by standard deviation calculated from the training set. These parameters were subsequently applied to the validation set to prevent data leakage. Categorical features, such as medium composition/usage and infant characteristics, were encoded for model interpretation.

Prior to model deployment, feature selection was conducted using a manual ablation strategy instead of automated algorithms. Some features, such business management information, post-culture examination results, and maternal blood tests results, were not incorporated into the final model. The preprocessed data were randomly split into a training dataset (80%) and an evaluation dataset (20%). Gradient-boosting-based models were developed using an XGBoost algorithm (version 3.0.0) because of its strong performance on structured data and ability to capture nonlinear relationships and feature interactions. Within the training dataset, key hyperparameters were optimized using a grid search strategy combined with stratified four-fold cross-validation. Specifically, the training data were further partitioned into four folds, where in each iteration, three folds were used for model training and one-fold for validation. The average performance across the four folds was used to identify the optimal hyperparameter configuration. For the culture duration model, the search space included the number of boosting rounds (n_estimators) (100-2,000), maximum tree depth (max_depth) (1-10), learning rate (eta) (0.001-0.2), subsampling ratios (subsample and colsample_bytree) (0.6-1.0), L1 regularization term (reg_alpha) (0.001-1.0) and L2 regularization term (reg_lambda) (1.0-2.0). In contrast, the cell viability model focused on a different search range to accommodate its specific learning dynamics, with “n_estimators” between 5,000 to 10,000, “max_depth” between 1 and 3, and “eta” between 0.001 and 0.01. All other hyperparameters were maintained at their default settings. After selecting the optimal hyperparameters based on cross-validation performance within the training dataset, the final model was retrained using the entire training dataset. Its predictive performance was then evaluated on the independent hold-out evaluation dataset to provide an unbiased estimate of model generalizability. This two-step evaluation framework was used to minimize overfitting and ensure robust estimation of model performance. The final optimized parameters selected for model training are detailed in Table 4.

### Establishment of XAI

XAI was implemented using SHAP approach to explain the contribution of each input parameter to UC-MSC quality. This method effectively provides a unified framework for quantifying the impact of individual parameters on model output 23. We used a SHAP summary plot to illustrate each parameter's positive or negative impact on the model outcome, culture duration, and cell viability. In the SHAP summary plot, the red dots represent groups associated with higher values or specific characteristics, whereas the blue dots indicate the opposite. Specifically, for birth weight, red denotes a higher weight; for cord blood type, delivery mode, infant sex, or operator, red represents the corresponding group identity. In the case of UC length and operation time, red indicates longer measurements. For medium composition (no additive), red represents nonuse; for a specific brand of a medium ingredient, red indicates the use of that brand. For surface antigen expression, red denotes high expression levels.

### Algorithm validation and analysis

Various metrics were used to evaluate model prediction performance. Sensitivity, specificity, and accuracy were assessed for a balanced view of the model's discriminative power. The area under the receiver operating characteristic curve (AUROC) was used as a global indicator of classification performance; an AUROC value close to 1.0 implies excellent discriminative power. The F1 score, which is the harmonic mean of precision (positive predictive value [PPV]) and recall (sensitivity), was calculated to account for imbalance and minimize both false positives and false negatives. The area under the precision-recall curve (AUPRC) was generated by plotting precision, also known as the PPV, against recall (sensitivity) across varying probability thresholds. The AUPRC summarizes the trade-off between precision and recall over all thresholds, with higher values indicating better performance. The model's confidence in its probabilistic predictions was determined using log loss, which penalizes incorrect predictions that are made with high confidence; a lower log loss indicates better model calibration. The Brier score was estimated as the mean squared difference between the predicted probabilities and the observed binary outcomes, providing an overall measure of probabilistic accuracy that reflects both calibration and discrimination. A lower Brier score indicates better predictive accuracy, with a value of 0 representing perfect predictions and 1 indicating the worst performance 24. Calibration curves were generated by grouping the predicted probabilities into deciles and plotting the observed event rates against the mean predicted probabilities per group, with perfect calibration corresponding to the 45° diagonal line.

### Sensitivity analysis

Sensitivity analysis was performed to evaluate the impact of adjustments in model cutoff values or specific features on predictive performance. The delta AUROC, which was the AUROC of the modified model minus that of the baseline model (ΔAUROC = AUROC_modified_ - AUROC_baseline_), was computed to quantify model performance changes. The stability of the top SHAP features was assessed by calculating the percentage of folds in which each feature consistently appeared.

## Results

**Figure 1:**
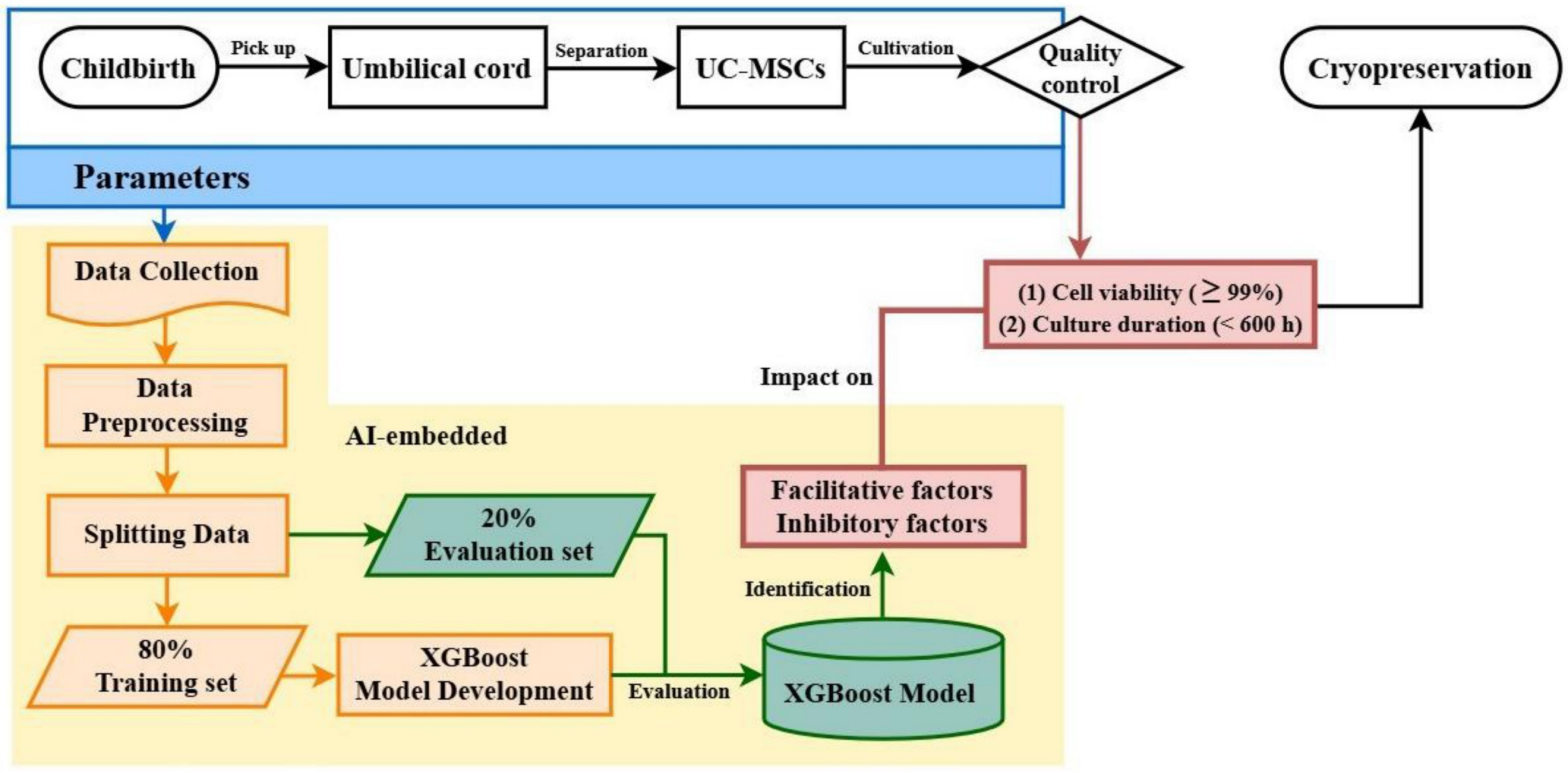
Diagram of model architecture. Machine learning model architecture for predicting suggested facilitative and inhibitory factors to optimize UC-MSC cell culture duration and cell viability.

We aimed to develop a pipeline using an XGBoost algorithm to identify the facilitatory and inhibitory factors influencing UC-MSC viability and culture duration (Fig. 1). We hypothesize that the studied parameters, originating from postpartum UC isolation to precryopreservation, may independently or interactively impact outcomes. Therefore, two criteria were separately adopted for quality evaluation: culture duration < 600 h and cell viability ≥ 99%. Parameters from childbirth, UC isolation, and UC-MSC cultivation were collected, preprocessed, and split into training and evaluation datasets. After model training and evaluation, the algorithm was used to identify the most influential factors that improve or degrade UC-MSC quality.

We selected 24 parameters that may influence the culture duration and viability of UC-MSCs (Table 1). Neonatal factors were adopted, such as birth weight, cord blood group and Rh type, infant sex, and original and processed navel cord lengths. The logistics/handling timelines from birth to UC-MSC isolation from the navel cord (called the birth-to-separation time) were recorded. This duration was further subdivided into four distinct time intervals: birth-to-pickup time, pickup-to-delivery time, delivery-to-processing time, and separation time. The birth-to-pickup time is the period the UC is stored at the hospital from childbirth until collection by the courier. The pickup-to-delivery time is the transportation period from the hospital to the processing facility. The delivery-to-processing time is the waiting period from the arrival of the UC at the processing facility to the start of its active laboratory handling. Finally, the separation time is the laboratory operation time required to isolate US-MSCs from UC tissue. The usage and brands of six culture medium components, total cell number, MSC-specific marker expression, and operators were also included in this study.

A total of 203 samples were collected. The median collection-to-precryopreservation duration was 564 h (interquartile range [IQR]: 107), with 67% of the samples meeting the required culture duration (< 600 h). The median cell viability was 98.9% (IQR: 0.9), and 50% of the samples (N = 101) exhibited ≥ 99% cell viability. Following the preset 80/20 split, 163 samples (80%) were allocated to the training set, and the remaining 40 samples (20%) constituted the model evaluation dataset.

**Figure 2:**
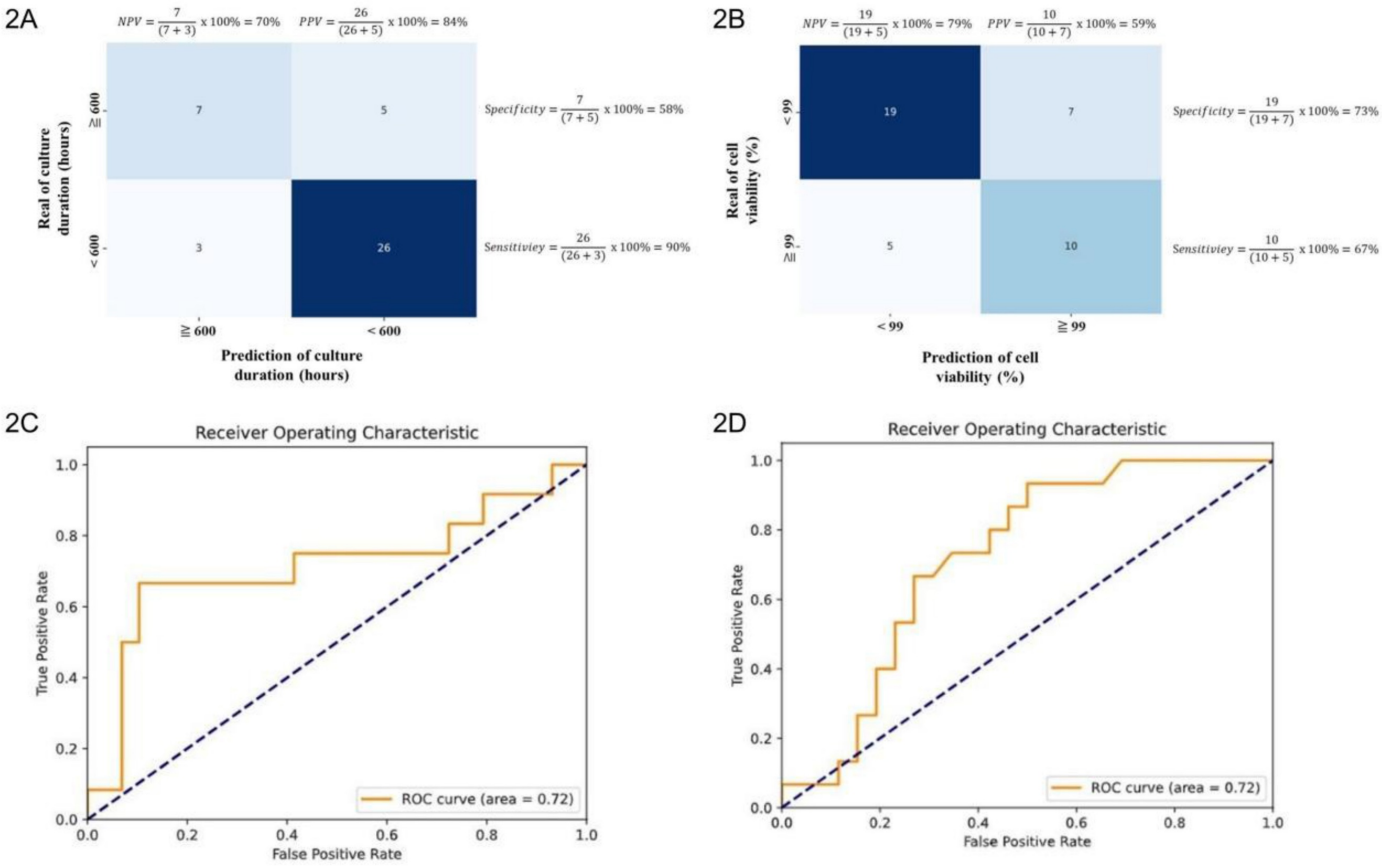
Performance evaluation of machine learning models. Two-way confusion matrix for the models in predicting proposed facilitative and inhibitory factors influencing (2A) cell culture duration and (2B) cell viability. AUROC curves illustrate the model's predictive ability for proposed facilitative and inhibitory factors affecting (2C) cell culture duration and (2D) cell viability.

Machine learning-driven analysis was used to identify parameter combinations associated with reductions in UC-MSC culture duration. The model yielded a sensitivity of 90% and a specificity of 58%, with a PPV of 84% and a negative predictive value (NPV) of 70% (Fig. 2A). The overall classification accuracy was 80%. Additionally, the AUROC was 0.72 (95% confidence interval [CI]: 0.53-0.89) (Fig. 2C), and the log loss was 0.55. The model achieved a sensitivity of 67% and a specificity of 73% in predicting cell viability. The PPV was 59%, and the NPV was 79% (Fig. 2B). The overall accuracy was 71%, with an AUROC of 0.72 (95% CI: 0.58-0.89) (Fig. 2D). The log loss was 0.63.

**Figure 3:**
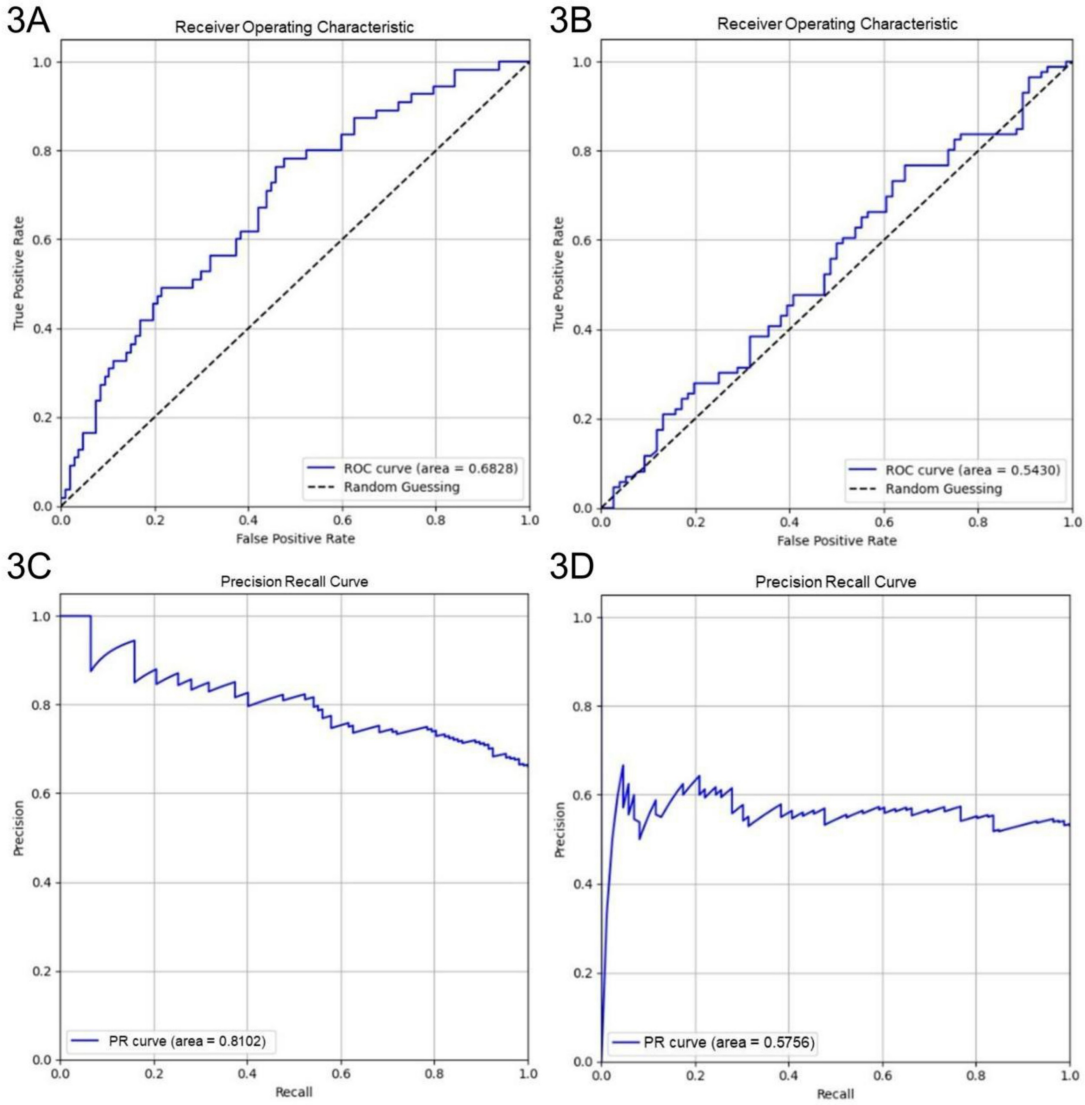
Performance of XGBoost models. Performance of XGBoost models showing AUROC and AUPRC for culture duration (3A and 3C) and cell viability (3B and 3D) from 4-fold cross-validation.

**Figure 4:**
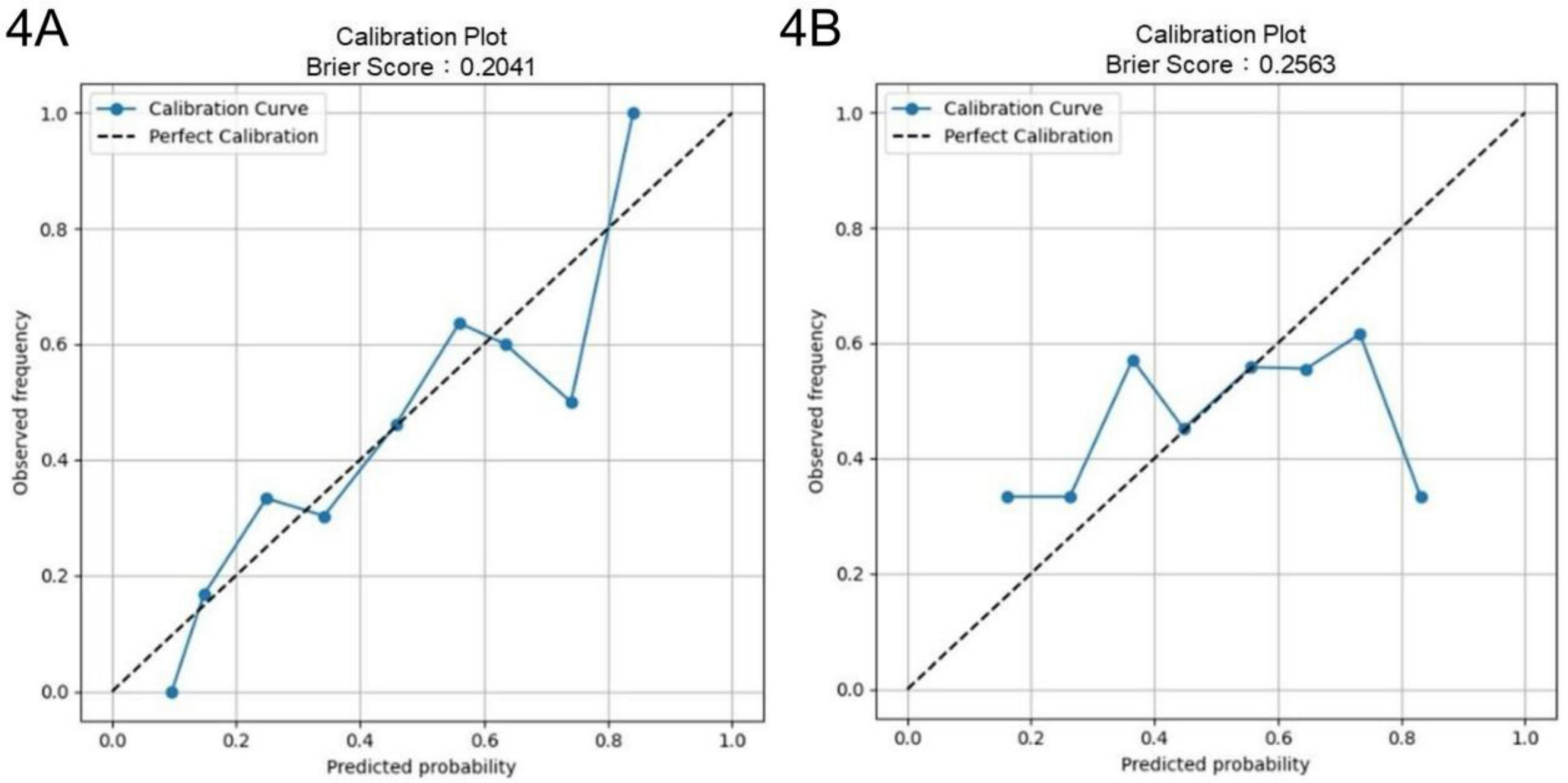
Brier score and calibration curves. Brier scores and calibration curves from 4-fold cross-validation for (4A) culture duration and (4B) cell viability models.

Four-fold cross-validation was performed to evaluate the generalization performance of the XGBoost model in identifying key parameters for optimizing culture duration and cell viability. The models achieved average accuracies of 0.69 and 0.54 in predicting shortened culture durations and higher cell viability, respectively. For predicting < 600 h culture durations, the culture duration model yielded an AUROC of 0.68 (95% CI: 0.60-0.77) and an AUPRC of 0.81 (95% CI: 0.73-0.88) (Figs. 3A and 3C). For predicting ≥ 99% cell viability, the AUROC was 0.54 (95% CI: 0.45-0.63), and the AUPRC was 0.58 (95% CI: 0.47-0.69) (Figs. 3B and 3D). The Brier score of the culture duration model was 0.20, and its calibration plot indicated that the predicted probabilities generally followed the observed frequencies, with some fluctuations at intermediate probability values (Fig. 4A). For the cell viability model, the Brier score was 0.26, and the calibration plot shows that the predicted probabilities closely matched the observed frequencies across most of the probability range, with only a slight overestimation at higher probabilities (Fig. 4B).

**Figure 5:**
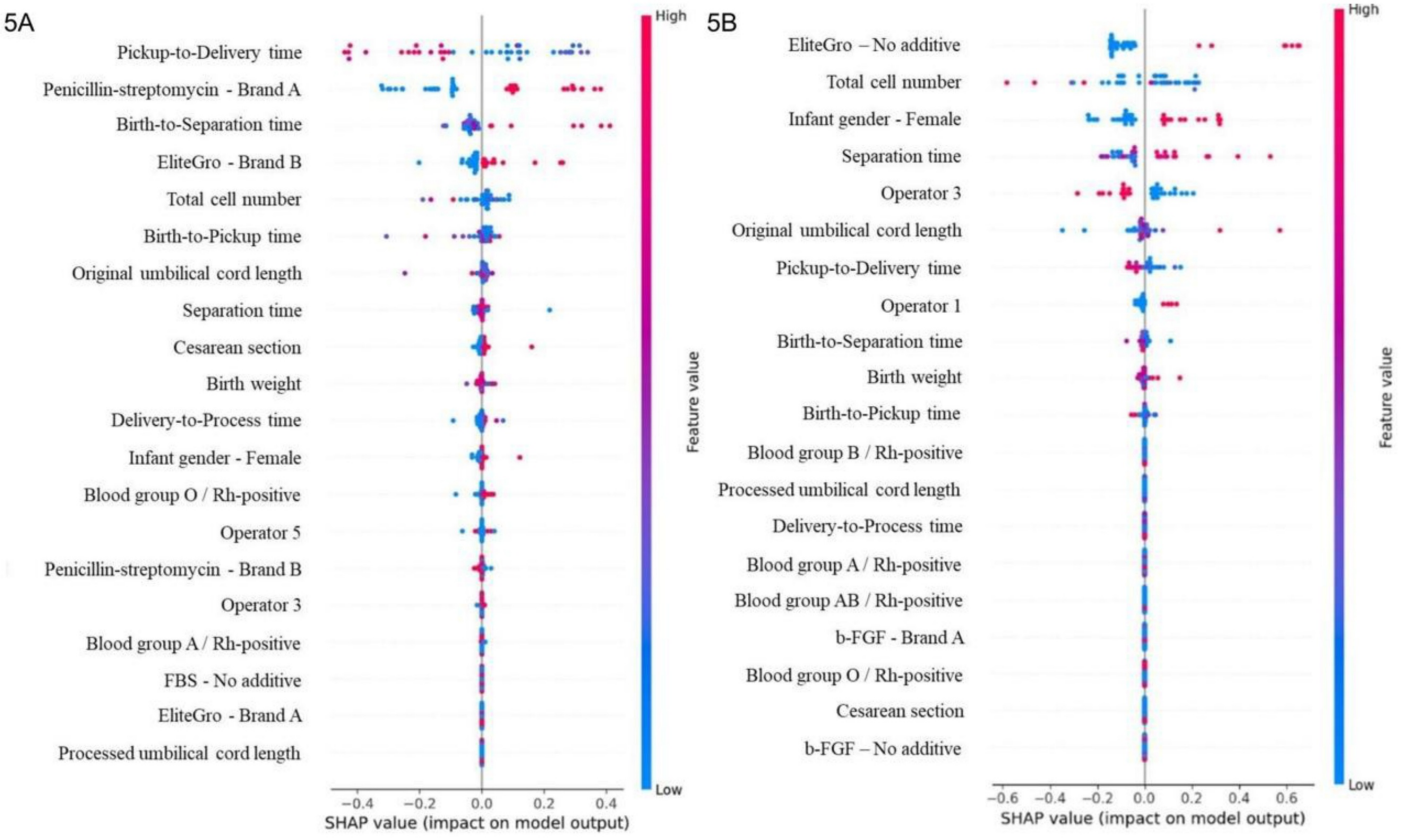
SHAP summary plot analysis. Ranking the importance of factors for (5A) shorter cell culture duration and (5B) higher cell viability. Positive SHAP values in (A) and (B) indicate that the corresponding feature values drive the model toward predicting a shorter cell culture duration and higher cell viability, respectively.

SHAP is a robust, widely adopted approach to interpreting complex ML models, offering detailed insights at global and local prediction levels. Figure 5 illustrates the magnitude of each parameter's contribution to the model's prediction and indicates its positive or negative effect on model outcomes. Several factors were found to be positively associated with short culture durations (< 600 h). Specifically, the use of penicillin-streptomycin (Brand A), prolonged birth-to-separation times, use of EliteGro (Brand B), and cesarean delivery showed positive SHAP values. Conversely, prolonged pickup-to-delivery times were negative associated, suggesting their role in extending culture times (Fig. 5A and Table 2). For samples exhibiting high cell viability (≥ 99%), SHAP highlighted distinct contributing parameters. Positive associations were observed with female neonatal sex and prolonged separation times, helping the model predict high cell viability. By contrast, the use of EliteGro, processing handling by Operator 3, and shorter original (at collection) UC lengths were negatively correlated with higher cell viability (Fig. 5B and Table 3). The detailed hyperparameters for the development of the XGBoost model are in Table 4.

Sensitivity analysis was conducted to evaluate the performance of the culture duration model at different classification thresholds. Adjusting the cutoff from 600 h to the median value of 564 h, thereby balancing group sizes, resulted in only a modest change in the AUROC (delta AUROC: 0.08; the formula: ΔAUROC = AUROC_culture duration < 564 h_ - AUROC_culture duration < 600 h_) but maintained 70% stability in the top 10 SHAP-ranked features. Notably, operator identity ranked fifth in SHAP feature importance for the cell viability model. Excluding this variable led to a minimal AUROC reduction (delta AUROC: 0.02). This finding suggests that the cell viability model's predictive structure was not primarily driven by operator identity alone, but rather by a set of top parameters.

## Discussion

In this study, we demonstrate that an interpretable XGBoost + SHAP framework can identify process-relevant drivers of two practical UC-MSC quality indicators, namely, culture duration and postprocessing viability, using routine manufacturing data. SHAP analysis clarified both the directionality and relative magnitude of feature effects, translating black-box predictions into bioprocess hypotheses that are testable and auditable in GMP-like (Good Manufacturing Practice) settings 20. Beyond confirming known influences, the model highlighted less-anticipated associations involving neonatal sex, specific medium components/brands, and operator-dependent variability, underscoring the value of multivariable, nonlinear modeling in cell manufacturing 7-13,17-18,20,25-27.

### Practical integration into manufacturing control

These interpretable outputs can be integrated immediately into quality systems in manufacturing. (1) Real-time dashboards and alerts: SHAP value streams can be visualized per batch to flag adverse drifts (e.g., prolonged pickup-to-delivery times) before they manifest as prolonged culture durations or viability declines. Thresholds can be embedded into line-clearance or batch-release checklists to trigger corrective actions (e.g., repacking, courier escalation, and temperature audit) 28,29. (2) Standard operating procedure (SOP) and medium standardization: feature attributions linked to specific medium components/brands or antibiotic use support targeted standardization and supplier qualification. Prior reports show that antibiotics can alter growth and gene expression; in this study, the identified associations between penicillin-streptomycin (brand specific) and shorter culture durations can influence brand-level evaluation, titration, or phased removal studies to balance sterility assurance and biological impact 30-34. Consistent with our observation, Li *et al.*, reported that specific concentrations of penicillin-streptomycin enhanced US-MSC proliferation and survival by regulating apoptosis-related gene expression 35, supporting the notion that penicillin-streptomycin plays an important role in UC-MSC culture duration. (3) Logistics engineering: the time partitions (birth-to-pickup, pickup-to-delivery, delivery-to-processing, and separation times) quantify where delays impair outcomes the most. Combining these with SHAP enables evidence-based changes (e.g., courier service level agreements, temperature controls, and redundancy in weekend coverage) that help stabilize transit-related risk 28,29. Prolonged transport or storage of UC prior to processing has been reported to reduce MSC proliferative 36, which is also observed in our analysis, showing that prolonged pickup-to-delivery time (SHAP Rank: 1) was linked to longer cell culture duration. (4) Operator training and competency: skill-dependent variations have long been recognized as a critical source of variability in cell manufacturing. However, excluding the operator identity in this study resulted in only a minimal reduction in AUROC for the cell viability model. It is important to note that operator identity should not be interpreted as an isolated causal determinant of cell viability. In cell manufacturing, operator-related effects often act as composite indicators that co-vary with other process parameters, such as processing timelines, task allocation, and medium usage, rather than reflecting on individual technical skills alone. Importantly, removal of operator variable did not materially alter model discrimination. This suggests that while individual expertise is essential, the variance captured in our dataset was likely overshadowed by more dominant parameters. Therefore, operator identity should be interpreted as a surrogate marker of execution-related process variability, highlighting opportunities for further SOP harmonization and workflow standardization rather than individual-level attribution.

### Biological and clinical interpretation

Female neonatal sex was positively associated with higher viability and slightly shorter culture duration in our dataset, complementing prior observations suggesting sex-linked differences in UC-MSC yields or properties 37-40. Within cell viability model, SHAP analysis revealed a consistently positive directional contribution of female neonatal sex across samples, suggesting that the model captured this variable as a stable trend-level signal. However, given the retrospective and observational nature of this study, these associations should be interpreted strictly as hypothesis-generating rather than as evidence of a direct biological mechanism. Further prospective and mechanistic studies are required to validate the physiological basis of sex-associated differences in MSC quality. While operator factors do have an impact on cell viability, their contributions were quietly smaller than that of neonatal sex and did not significantly alter the overall positive effect observed for female-derived cells, suggesting this influence is independent of operator variability. The association between cesarean delivery and reduced times to early harvest echoes standardized UC-MSC isolation work suggesting that the perinatal context may influence early outgrowth and handling efficiency 41. Collectively, these findings generate mechanistic hypotheses that can be examined in controlled prospective studies (e.g., secretome differences between sexes, peripartum hormonal milieu, and tissue stress between delivery modes).

### Generalizability to other cell sources and settings

Given comparable process metadata, this source-agnostic framework can be retrained on other mesenchymal sources (adipose-derived and bone-marrow MSCs) or adjacent products. Many variables are shared, such as logistics, medium compositions, and operator factors, whereas SHAP reveals source-specific drivers to inform tailored SOPs. Similarly, the proposed approach can be scaled to medium-optimization cycles by coupling active-learning designs with model explanations to prioritize informative experiments 17,25,27.

### Model performance, validation, and limitations

Model discrimination for both endpoints (AUROC: 0.72) is modest to good for retrospective (Figs. 2C and 2D), heterogeneous manufacturing data and remains critically interpretable. In external cross-validation, the culture duration model exhibited moderate discrimination (AUROC: 0.68), with strong probability calibration (Brier score: 0.20) and an AUPRC of 0.81 (Figs. 3A, 3C, and 4A). Notably, although the culture duration dataset exhibited moderate class imbalance (136 vs 67), class weighting strategies, the scale_pos_weight parameter, were evaluated during hyperparameter tuning. Since this did not yield meaningful improvements in AUROC or F1 score, the final model was trained without explicit weighting to maintain model simplicity. This indicates the culture duration model's reliability in identifying key factors at quality-relevant thresholds. The cell viability model displayed a slightly lower AUROC (AUROC: 0.54), a result that remained consistent across all cross-validation folds. This stability suggests that modest performance origins from the intrinsic variability or noise inherent in the viability endpoint rather than model instability, particularly as the outcome classes were naturally balanced (102 vs 101), requiring no imbalance correction. Given this modest discriminative performance, the cell viability model is not intended for standalone prediction or batch-level decision making. Accordingly, SHAP-derived feature importance should be interpreted as reflecting consistent directional associations and process-level trends rather than precise causal effects. Prior studies have shown that global feature attributions can remain informative for process understanding and hypothesis generation even when point-prediction accuracy is constrained by noisy or highly clustered biological endpoints 42. While we incorporated key surface markers (CD73, CD90, and CD105) of UC-MSCs quantified via flow cytometry, their contributions to the model were not significant, as evidenced by their SHAP values 0.0, 0.0, and 0.1, respectively. This indicates that cell viability may be governed by complex biological or experimental factors not fully captured by the current feature set. Previous studies have highlighted that metabolomic profiling, such as tracking glucose metabolism, pyruvate catabolism, and amino acid dynamics, may provide a higher resolution and more dynamic representation of MSC bioenergetics and functional states that are not captured by conventional surface markers 43. Similarly, incorporating a broader panel of biomarkers, including lineage-associated gene expression profiles or additional surface antigens, may better reflect the biological heterogeneity and therapeutic potential of UC-MSCs 44. Although beyond the scope of the present retrospective dataset, integrating such metabolomic features and expanded CD marker panels into future machine-learning frameworks represents an important direction for improving predictive performance and deepening mechanistic insight 43-45. Despite this slightly lower AUROC, the model maintained acceptable calibration (Brier score: 0.26), with only mild overestimation at higher predicted probabilities (Figs. 3B and 4B), indicating its output remained interpretable for quality-control decisions. These results highlight the value of evaluating both differentiation and calibration, as well-calibrated probabilities are critical for predictive models in manufacturing and batch-release contexts.

Sensitivity analysis demonstrated that the culture duration model was robust to threshold variations, with only a modest change in the AUROC and 70% stability in the top 10 SHAP-ranked features, when the cutoff was adjusted from 600 h to 564 h. Therefore, the model predicted underlying biological and manufacturing parameters rather than artifacts of threshold selection. The stable features enhance confidence in leveraging these artificial intelligence-derived insights to guide process optimization under varying operational definitions. Threshold-based testing for cell viability was not conducted because the current cutoff (≥99%) was near the median and had a narrow IQR. The near-ceiling distribution presents a potential challenge for model learning, potentially explaining the limited predictive performance for this specific outcome. Although dichotomization using predefined thresholds is operationally meaningful in a manufacturing context, converting highly clustered continuous data into a binary outcome may reduce information content and statistical power. In the present study, we focused on binary classification to align with existing quality-control thresholds; however, future research could explore alternative labeling strategies, such as treating cell viability as a continuous regression target, which may better preserve granular variance and enhance model learning for bioprocess monitoring.

To enhance generalizability, future work should expand prospective data capture, include site-to-site variations, and incorporate cross-validation/bootstrapping stability checks for feature ranking. Limitations include single-facility bias, incomplete capture of potential confounders inherent to operational datasets, and the retrospective study design 21,28,29,41. Nonetheless, the real-world nature of the examined data increases the practical relevance of the results and establishes a deployable foundation for closed-loop, data-driven quality control.

## Conclusions

We developed and validated an interpretable ML framework that can be used to identify key determinants of UC-MSC viability and culture duration. Our findings highlight that neonatal factors, processing timelines, medium composition, and operator-related variability jointly influence UC-MSC quality. Specifically, the culture duration model exhibits robust performance, whereas the cell viability model functions as a tool for identifying the meaningful trends and contribution factors. By providing transparent feature attribution, the framework offers actionable insights for process control, SOP refinement, and operator training. The framework's scalability will allow for its application across different cell sources and manufacturing settings, supporting data-driven optimization and enhancing product consistency. Our approach is a scalable, data-driven solution for quality management in regenerative cell manufacturing.

## Figures and Tables

**Table 1:** Characteristics of machine-learning parameters.

Characteristic	Culture Duration Group		Cell Viability Group	
	Short term (< 600 h)	Long term (≥ 600 h)	High viability (≥ 99%)	Low viability (< 99%)
**Birth weight (g)**				
≤ 2000	3	0	2	1
2001-3,000	64	26	48	42
3,001-4,000	67	40	50	57
> 4,000	2	1	1	2
**Cord blood grouping / RH typing**				
A / Rh-positive	33	21	25	27
B / Rh-positive	32	12	22	22
O / Rh-positive	61	32	47	46
AB / Rh-positive	6	1	3	4
N/A	4	1	4	1
**Delivery mode**				
Cesarean section	57	29	42	44
Vaginal birth	79	38	59	58
**Infant sex**				
Male	66	35	45	56
Female	70	32	56	46
**Original UC length (cm)**				
≤ 20	4	6	3	7
21-30	61	24	30	45
31-40	49	28	40	37
41-50	16	6	11	11
> 50	6	3	7	2
**Processed UC length (cm)**				
≤ 20	120	54	85	89
21-30	14	12	15	11
31-40	2	1	1	2
**Operator**				
Operator 1	28	7	22	13
Operator 2	37	16	26	27
Operator 3	41	18	22	37
Operator 4	8	7	6	9
Operator 5	22	19	25	16
**Birth-to-pickup time (h)**				
≤ 1.00	12	5	9	8
1.01-5.00	80	45	62	63
5.01-10.00	22	13	18	17
10.01-20.00	22	3	11	14
> 20.01	0	1	1	0
**Pickup-to-delivery time (h)**				
≤ 1.00	21	3	11	13
1.01-5.00	36	22	36	22
5.01-10.00	31	10	19	22
10.01-20.00	39	27	28	38
> 20.01	9	5	7	7
**Delivery-to-processing time (h)**				
≤ 1.00	75	37	59	53
1.01-5.00	45	23	33	35
5.01-10.00	10	5	5	10
10.01-20.00	5	2	4	3
> 20.01	1	0	0	1
**Separation time (h)**				
≦ 10.00	33	24	29	28
10.01-20.00	39	20	32	27
20.01-30.00	64	22	40	46
> 30.01	0	1	0	1

**Birth-to-separation time (h)**				
≤ 20.00	5	5	7	3
20.01-30.00	56	27	43	40
30.01-40.00	38	20	26	32
40.01-50.00	33	13	22	24
> 50.01	4	2	3	3
**Penicillin-streptomycin**				
No additive	3	2	0	5
Brand A	75	14	39	50
Brand B	58	51	62	47
**FBS**				
No additive	110	37	60	87
Brand A	26	30	41	15
**0.5% Trypsin**				
No additive	110	37	60	87
Brand A	26	30	41	15
**0.05% Trypsin**				
No additive	109	37	59	87
Brand A	27	30	42	15
**b-FGF**				
No additive	82	18	41	59
Brand A	54	49	60	43
**EliteGro**				
No additive	26	30	41	15
Brand A	76	24	39	61
Brand B	34	13	21	26
**TrypLETM Select (10X)**				
No additive	29	30	42	17
Brand A	107	37	59	85
**TrypLETM Select (1X)**				
No additive	26	30	40	16
Brand A	110	37	61	86
**Total cell number (× 10^7^)**				
≤ 10.00	90	36	65	61
10.01-20.00	29	18	26	21
20.01-30.00	4	6	7	3
> 30.01	11	6	2	15
N/A	2	1	1	2
**CD73 expression (%)**				
98.0-98.9	1	2	1	2
99.0-99.9	68	22	44	46
100	63	43	56	50
N/A	4	0	0	4
**CD90 expression (%)**				
96.0-97.9	4	0	1	3
98.0-98.9	2	2	2	2
99.0-99.9	82	33	60	55
100	44	32	38	38
N/A	4	0	0	4
**CD105 expression (%)**				
94.0-95.9	5	4	3	6
96.0-97.9	12	10	8	14
98.0-98.9	22	11	20	13
99.0-99.9	87	41	67	61
100	6	1	3	4
N/A	4	0	0	4

**Table 2:** Facilitatory and inhibitory factors for optimization for shorter cell durations.

Facilitatory	Inhibitory
Use of penicillin-streptomycin (Brand A) Prolonged birth-to-separation time Use of EliteGro (Brand B) Cesarean section	Prolonged pickup-to-delivery time

**Table 3:** Facilitatory and inhibitory factors for optimization for higher cell viability.

Facilitatory	Inhibitory
Infant sex (female) Prolonged separation time	Use of EliteGro Operator 3 Shorter original UC length

**Table 4:** Tuned hyperparameters for XGBoost.

Training parameter		Culture duration	Cell viability
**Random Seed**		42	42
**XGBoost version**		3.0.0	3.0.0
XGBoost hyperparameters*	**n_estimators**	253	8,000
	**max_depth**	5	1
	**eta**	0.03	0.001
	**subsample**	0.9614	1 (default)
	**colsample_bytree**	0.7477	1 (default)
	**reg_alpha**	0.618	0 (default)
	**reg_lambda**	1.908	1 (default)
**Class imbalance**		none	none
**Missing value strategy**		XGBoost (default)	XGBoost (default)
**Preprocessing**		standard scaler and one-hot encoder	standard scaler and one-hot encoder

* The remaining hyperparameters were kept at XGBoost defaults, and hyperparameters were tuned before cross-validation.

## Data Availability

The datasets generated or analyzed during this study are available from the corresponding author on reasonable request.

## Abbreviations

AUPRC: Area under the precision-recall curve
AUROC: Area under the receiver operating characteristic curve
GMP: Good Manufacturing Practice
ML: Machine learning
MSC: Mesenchymal stem cell
PPV: Positive predictive value
SHAP: Shapley additive explanation
SOP: Standard operating procedure
UC: Umbilical cord
XAI: Explainable artificial intelligence
XGBoost: Extreme gradient boosting
